# Genome-Wide Analysis of Simple Sequence Repeats and Efficient Development of Polymorphic SSR Markers Based on Whole Genome Re-Sequencing of Multiple Isolates of the Wheat Stripe Rust Fungus

**DOI:** 10.1371/journal.pone.0130362

**Published:** 2015-06-12

**Authors:** Huaiyong Luo, Xiaojie Wang, Gangming Zhan, Guorong Wei, Xinli Zhou, Jing Zhao, Lili Huang, Zhensheng Kang

**Affiliations:** 1 College of Plant Protection, Northwest A&F University, Yangling, Shaanxi, People's Republic of China; 2 State Key Laboratory of Crop Stress Biology for Arid Areas, Yangling, Shaanxi, People's Republic of China; USDA-ARS-SRRC, UNITED STATES

## Abstract

The biotrophic parasitic fungus *Puccinia striiformis* f. sp. *tritici* (*Pst*) causes stripe rust, a devastating disease of wheat, endangering global food security. Because the *Pst* population is highly dynamic, it is difficult to develop wheat cultivars with durable and highly effective resistance. Simple sequence repeats (SSRs) are widely used as molecular markers in genetic studies to determine population structure in many organisms. However, only a small number of SSR markers have been developed for *Pst*. In this study, a total of 4,792 SSR loci were identified using the whole genome sequences of six isolates from different regions of the world, with a marker density of one SSR per 22.95 kb. The majority of the SSRs were di- and tri-nucleotide repeats. A database containing 1,113 SSR markers were established. Through *in silico* comparison, the previously reported SSR markers were found mainly in exons, whereas the SSR markers in the database were mostly in intergenic regions. Furthermore, 105 polymorphic SSR markers were confirmed *in silico* by their identical positions and nucleotide variations with INDELs identified among the six isolates. When 104 *in silico* polymorphic SSR markers were used to genotype 21 *Pst* isolates, 84 produced the target bands, and 82 of them were polymorphic and revealed the genetic relationships among the isolates. The results show that whole genome re-sequencing of multiple isolates provides an ideal resource for developing SSR markers, and the newly developed SSR markers are useful for genetic and population studies of the wheat stripe rust fungus.

## Introduction

Wheat stripe (yellow) rust is a devastating disease causing calamitous production losses of wheat, one of the most important cereal crops worldwide [1], endangering global food security. It is caused by the obligate biotrophic parasitic fungus *Puccinia striiformis* f. sp. *tritici* (*Pst*) and occurs in most wheat areas with cool and moist weather conditions during the growing season [2]. *Pst* requires living host (wheat/grasses and Berberis/Mahonia spp.) to complete the asexual and sexual phases of its life cycle [3]. Its dikaryotic urediniospores are the main spores that infected wheat and are able to spread via the wind up to thousands of kilometers from the initial infection sites [4–6]. *Pst* populations often possess a high genetic diversity and virulence variability [7], giving them the ability to circumvent specific resistance genes incorporated in wheat cultivars within only a few years after release [5,8,9]. Understanding the mechanisms of the pathogen diversity and virulence variability is important for control of the disease.

Simple sequence repeats (SSRs), also known as microsatellites, are tandem repeats of 2–6 base pairs of DNA flanked by sequences that are generally unique in the genome, but conserved in the organism populations [10,11]. The unique flanking sequences may provide templates that facilitate the development of specific primers for amplifying SSR alleles by polymerase chain reaction (PCR). Allelic differences identified in this manner indicate variable numbers of repeat units present at SSR loci. SSRs have been used as molecular markers in many organisms because they are abundant, highly polymorphic and repeatable. Because SSR markers are often co-dominant, they are preferred over dominant markers for genetic studies of diploid or higher ploidy organisms [12–19].

For *Pst*, SSR markers have been developed using microsatellites enrichment DNA library [20] and expression sequence tag (EST) libraries [21–23], as well as genomic sequences [24], and are the primary molecular markers used in genetic and population studies. For example, SSR markers were used to reveal the existence of the sexual and parasexual cycles [25–27], estimate the genetic diversity of different *Pst* populations [28–31] and analyze the origin, migration routes and worldwide population structures of *Pst* [32]. However, only a small number of SSR markers were used in these studies. For more sophisticated studies, more SSR markers are needed.

Whole-genome sequence of multiple isolates can provides data for developing genetic markers. The first reported *Pst* genome is that of isolate PST-130 in the USA, which was obtained through next generation sequencing [33]. Bailey et al. [24] reported 25 SSR markers derived from the sequences of this isolate. The genome of the CYR32, one of the most dominant *Pst* races in China, was assembled using a 'fosmid-to-fosmid' strategy to reduce the affect of genome heterozygosity [34]. The assembled genome of CYR32 (~110 Mb) was larger than the draft genome of PST-130 (~64.8 Mb), but was comparable with the genome of PST-78 (~117Mb), another sequenced isolate from the USA (http://www.broadinstitute.org, unpublished). The genome assembly of CYR32 is suitable for the genome-wide analysis of SSRs.

In this study, we identified 4,792 SSR loci from the CYR32 genome sequence and determined the distribution patterns of SSRs in this isolate and five additional isolates from different countries. To provide the *Pst* community with more markers for more sophisticated studies, we developed a SSR marker database containing 1,113 SSR markers based on the genomes of the six isolates. We also validated 82 polymorphic SSR markers.

## Materials and Methods

### 2.1 *Pst* Isolates

The sequence reads of six *Pst* isolates, 104E137A from Australia, CYR23 and CYR32 from China, Hu09-2 from Hungary, PK-CDRD from Pakistan and PST-78 from the USA, were used to identify polymorphic SSR loci [34]. For SSR marker validation, 21 isolates collected from the USA, Hungary and China were used (Table 1). Urediniospores were multiplied on wheat from a single urediniospore for each isolate and used for DNA extraction.

**Table 1:** Description of the *Puccinia striiformis* f. sp. *tritici* isolates used to evaluate the polymorphism of SSR markers.

**ID**	**Isolates**	**Geographic origin**
**1**	PST-1	USA
**2**	PST-17	USA
	PST-43	USA
**4**	PST-78	USA
**5**	PST-114	USA
**6**	XIONG	Hungary
**7**	CYR20	China
**8**	CYR23	China
**9**	CYR31	China
**10**	CYR32	China
**11**	HA-7	Xinjiang, China
**12**	G148-2	Xinjiang, China
**13**	G149-2	Xinjiang, China
**14**	G150-14	Xinjiang, China
**15**	G164-26	Xinjiang, China
**16**	G167-10	Xinjiang, China
**17**	G167-12	Xinjiang, China
**18**	G167-14	Xinjiang, China
**19**	GL17-2	Gansu, China
**20**	DT-6	Qinghai, China
**21**	G862-34	Tibet, China

### 2.2 *Pst* genome sequences and INDEL discovery

The previously published genome sequence of CYR32 [34] was used as the reference genome. Sequence reads used in this study were obtained from PE (paired ends) libraries with 500bp inserts using the Illumina whole genome sequencing technology. High quality reads were extracted with NGSQCToolkit_v2.3.3 [35], SolexaQA_v.2.2 [36] and FASTX_Toolkit (http://hannonlab.cshl.edu/fastx_toolkit/) software, and were aligned against the CYR32 reference genome using BWA-0.7.9a [37] software. The GATK [38] duplicate removal, INDEL (insertions or deletions) realignment and base quality score recalibration, and performed INDEL discovery across all six isolates were conducted simultaneously according to GATK Best Practices recommendations [39,40]. The average re-sequencing depth was 18.98, and genome coverage was 95.52%. A consensus genome sequence for each isolate was generated using Samtools-0.1.19 [41] software. The joint INDEL discovery across all six isolates was performed using Samtools-0.1.19. Only concordance INDELs, which were discovered by both methods [42] and passed a quality filter (QD>2.0, FS<200.0, ReadPosRankSum>-20.0) [39,40], were used to identify candidate polymorphic SSR loci.

### 2.3 SSR identification and primer design

The flow chart presented in supporting information S1 Fig illustrates the main steps used to develop SSR markers. SSR motifs were identified in the six genomes using a MISA script downloaded from http://pgrc.ipkgatersleben.de/misa/. Only perfect SSRs, including di-, tri-, tetra-, penta-, and hexa-nucleotide motifs with numbers of uninterrupted repeat units greater than 7, 6, 5, 4, and 4, respectively, were included in this study.

In order to identify SSR loci with unique flanking sequences, repeat motifs and 300bp flanking sequences on each side were used for BLASTN search against the genomic sequences (e-value cut-off of 1e-10), and filtered with >90% identity and >85% alignment length of the flanking sequences. SSR loci with a single hit were identified as candidate loci for marker development. Primer pairs of SSR markers were designed using Primer 3 software (http://primer3.sourceforge.net/) with the following parameters: minimum, maximum, and optimal sizes were 18, 27, and 20 nt, respectively; minimum and maximum GC content were 20% and 80%, respectively; minimum, maximum, and optimal Tm were 57, 63, and 60°C, respectively; and product size range was from 100 to 300 bp.

The specificity of designed primers was confirmed through electronic polymerase chain reaction (E-PCR) [43,44] in the genomes using following parameters: the word size was 9, the discontinuous word was 1, the maximal deviation of observed product size to expected size was 100, both the number of mismatches and gaps allowed per primer were 1. The 84 previously published *Pst* SSR markers [20–24] were downloaded and amplified by E-PCR for comparison.

In addition, the genomic locations of SSRs were annotated. The exon, intron, and intergenic region were determined based on the original annotation of the CYR32 reference genome. The promoter regions were designated at 2 kb upstream of the start site of first exon. A protein was designated “secreted” if it contained a signal peptide and “pathogenicity-related” if it showed high similarity to a protein in the PHI database [45,46]. Moreover, a custom Perl script was used to directly identify polymorphic SSRs by the identical sequence position and nucleotide variation of identified INDELs and of developed SSR markers.

### 2.4 Experimental validation of polymorphic SSR markers

To experimentally validate putative polymorphic SSR markers, 104 primer pairs were synthesized by Sangon Biotech (Shanghai) Co., Ltd. Twenty-one *Pst* isolates in Table 1 were used in this experiment. Genomic DNA was extracted from urediniospores using a Fungal gDNA Kit (Biomiga). PCR reactions were performed in a 25μl volume containing 2.0μl template DNA (50ng/μl), 2.5μl reaction buffer (10×, Mg2+ free), 2.0μl Mg2+ (25mM), 2.0μl dNTPs (2.5mM), 1μl each primer (10mM), 0.2μl Taq DNA polymerase (5U/μl, Thermo Scientific) and 14.3μl ddH_2_O. The PCR conditions were as follows: 95°C for 4min; 35 cycles of 94°C for 45s, 55–58°C (varies for each primer pair) for 45s, and 72°C for 45s; and 72°C for 10min. PCR products were transferred directly from the thermocycler to the load tray of the Qiaxcel system. Separation was performed using the default OM500 method following the manual of QIAxcel DNA High Resolution Kit. The product sizes were automatically calculated in base pairs (bp), and gel views were exported using the QiaxcelScreenGel software. The number of alleles was recorded, and the polymorphism information content (PIC) was calculated. Statistical analysis was conducted by POPGENE version 1.32 software. Principal component analysis was conducted to show relationships of the 21 isolates based on genotypes of 82 polymorphic SSR markers using the NTSYSpc program [47]. A similarity matrix based on Dice coefficient was also generated using the SIMQUAL module in the NTSYSpc. Cluster analysis was conducted with the UPGMA (Unweighted pair-group method, arithmetic average) method in the SAHN module of the NTSYSpc. The COPH and MXCOMP modules were used to choose the dendrogram with the best fit to the similarity matrix. Robustness of branches of the dendrogram was determined by bootstrap analysis with the Winboot program [48].

## Results

### 3.1 The abundance of SSRs in the Pst genome

A total of 4,792 unique SSR loci were identified by searching through the genomes of the six *Pst* isolates from five countries with the MISA script. The number of SSRs varied among the different isolates (Table 2), ranging from 4,536 in PST-78 to 4,665 in CYR32 with the average of 4,576. Of the total of unique SSRs, 4,310 were common in all six isolates, and 492 in one or more, but not all isolates. Of the repeat motifs observed, the di-nucleotide motif was the most abundant (46.87%), followed by tri- (32.32%), penta- (9.39%), hexa- (5.95%), and tetra- (5.47%) nucleotide motifs (Table 3). The average intervals for di-, tri-, tetra-, penta-, and hexa-nucleotide SSRs were 51.04, 74.32, 445.34, 257.61, and 411.99 kb, respectively (Table 2). Considering the total of 4792 loci, the average interval was estimated as 22.95 kb, indicating the high abundance of SRRs in the *Pst* genome.

**Table 2:** Numbers and density of the SSR loci identified in the genomes of six *Puccinia striiformis* f. sp. *tritici* isolates.

	SSR numbers						SSR interval (kb)					
Isolates	DNR*	TNR*	TTR*	PNR*	HNR*	Total	DNR*	TNR*	TTR*	PNR*	HNR*	Total
**CYR32**	2211	1510	257	424	263	4665	49.75	72.85	428.02	259.43	418.25	23.58
**CYR23**	2175	1471	248	429	273	4596	50.57	74.78	443.55	256.41	402.93	23.93
**PK-CDRD**	2147	1487	244	426	271	4575	51.23	73.97	450.82	258.22	405.90	24.04
**104E137A**	2134	1475	245	425	264	4543	51.55	74.58	448.98	258.82	416.67	24.21
**Hu09-2**	2138	1467	242	429	267	4543	51.45	74.98	454.55	256.41	411.99	24.21
**PST-78**	2124	1469	246	432	265	4536	51.79	74.88	447.15	254.63	415.09	24.25
**Average**	2155	1480	247	427	267	4576	51.04	74.32	445.34	257.61	411.99	24.04
**Total**	2246	1549	262	450	285	4792	48.98	71.01	419.85	244.44	385.96	22.95
**Common**	2039	1410	228	391	242	4310	53.95	78.01	482.46	281.33	454.55	25.52

* DNR, TNR, TTR, PNR, and HNR indicate di-, tri-, tetra-, penta-, and hexa-nucleotide SSRs, respectively.

**Table 3:** Numbers and percentages of the identified SSR loci and developed SSR markers

	Total identified SSR loci		Newly developed SSR markers		105 polymorphic SSR markers	
Motifs	Number	Percentage (%)	Number	Percentage (%)	Number	Percentage (%)
**DNR** *	2246	46.87	563	50.58	70	66.67
**TNR** *	1549	32.32	305	27.40	23	21.90
**TTR** *	262	5.47	84	7.55	4	3.81
**PNR** *	450	9.39	99	8.89	4	3.81
**HNR** *	285	5.95	62	5.57	4	3.81
**Total**	4792	100.00	1113	100.00	105	100.00

* DNR, TNR, TTR, PNR, and HNR indicate di-, tri-, tetra-, penta-, and hexa-nucleotide SSRs, respectively.

**Fig 1:**
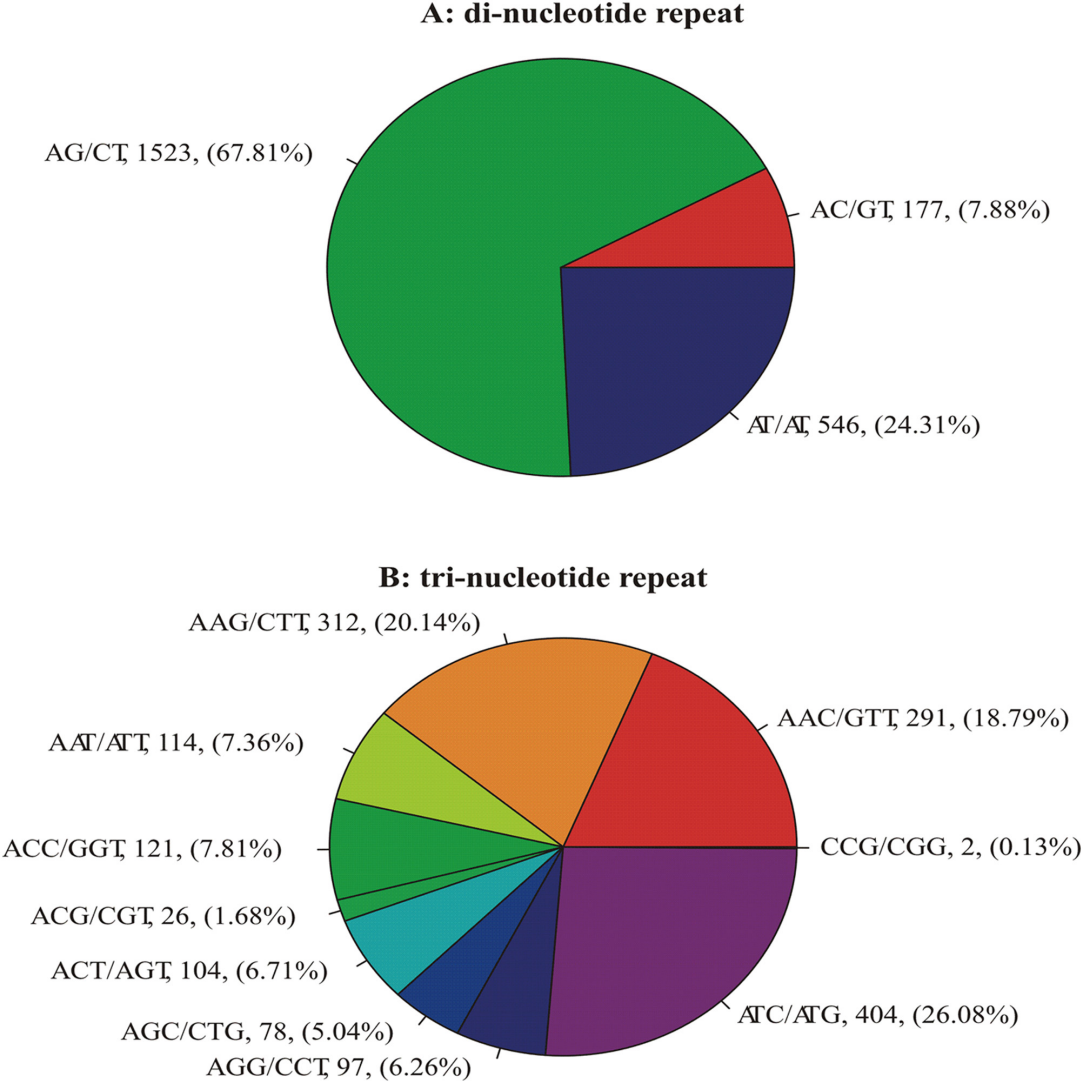
Percentage of different repeat types of di- and tri-nucleotide SSRs in isolate CYR32 of *Puccinia striiformis* f. sp. *tritici*

The SSRs were found to be 170 repeat types (S1 Table), some of which were dominant. Of the three di-nucleotide motifs (Fig 1A), AG/CT was the most abundant (1523, 67.81%) followed by AT/AT (546, 24.31%), while AC/GT was the least frequent (177, 7.88%). Of the ten types of tri-nucleotide motifs (Fig 1B), ATC/ATG was the most frequent (404, 26.08%), followed by AAG/CTT (312, 20.14%) and AAC/GTT (291, 18.79%), while other seven types were lower in frequency (2–121, 0.13–7.81%). There were 22 types of tetra-nucleotide motifs, of which the top three types (AAAT/ATTT, AAAG/CTTT and AAAC/GTTT) accounted for 20.99% (55), 19.47% (51) and 14.50% (38), respectively, while other types ranged from 0.38% (1) to 7.63% (20). Of the 53 types of penta-nucleotide motif, AAAAC/GTTTT (68, 15.11%), and AAAAG/CTTTT (53, 11.78%) were the most common, while others ranged from 0.22% (1) to 9.78% (44). ACACTC/AGTGTG was the most common repeat type (39, 13.68%) of the 82 types of hexa-nucleotide motifs, and others ranged from 0.35%(1) to 6.67%(19). In addition, the AG/CT di-nucleotide repeat unit had the highest repeat number (40) of all identified SSRs. The average repeat lengths varied among repeat types, ranging from 15.32bp for AC/GT to 51.00bp for AAGGAG/CCTTCT.

### 3.2 Screening for SSR candidate loci and developing Pst SSR markers

In order to identify candidate loci for marker development, all 4792 SSR motifs, together with their 300bp flanking sequences on each side were used for a BLASTN search against the genomic sequence. 1,446 loci (30.18%) produced single hits based on our cutoff across all six *Pst* isolates. Primers were designed for the 1,446 candidate loci with Primer3 software, and the specificity of these primers was validated *in silico* using E-PCR software, which result in 1113 primer pairs "amplified" expected bands and the remaining 333 "amplified" multiple bands or produced false matches (S2 Table). The 1113 SSR markers with specific primers form a new database of *Pst* SSR markers. This database accounts for 23.23% of the total 4792 identified loci (Table 3). In the database, SSRs with di-, tri-, tetra-, penta-, and hexa- nucleotide motifs accounted for 50.58%, 27.40%, 7.55%, 8.89%, and 5.57% of the SSRs, respectively (Table 3).

### 3.3 In silico comparison with previously reported SSR markers

A total of 84 SSR markers had been reported for *Pst* (S3 Table) prior to the present study. Through E-PCR, 34 SSR markers were found to have unique primer binding sites (S2B Fig), 23 had two primer binding sites (S3B Fig), 5 had three primer binding sites (S4A Fig), and the remaining 22 markers had no proper primer binding sites (S4 Table). The 34 SSR markers with unique binding sites "amplified" 30 SSR loci in 29 scaffolds in the *Pst* genome (S2B Fig). Among the 30 SSR loci, 14 loci also could be amplified with 14 newly developed SSR markers, as shown in S2C Fig. In addition, we found that markers CPS02, CPS10, CPS13, and PstP001 mapped to the same loci as RJ3N, RJ8N, RJ13N, and Pst002, respectively (S2B Fig). These results should be helpful in selecting SSR markers by reducing the possibility of using "different" primers to amplify the same SSR loci.

### 3.4 Distribution of SSR markers in different genomic regions

In order to investigate the distribution of SSR markers among different genomic regions, we mapped them to exon, intron, intergenic and promoter regions based on the original annotations of the CYR32 reference genome (Table 4). A high percentage of markers in our SSR marker database were located in intergenic region (39.26%), followed by promoter region (27.94%) and exon (20.13%), while those in intron were the fewest(12.67%). The 30 previously reported SSR markers were found mostly in exon (66.67%), because most were identified from EST libraries. Therefore, the newly identified SSR markers should provide more information in non-transcribed regions of the *Pst* genome.

**Table 4:** Distribution of SSR markers in different genomic regions of *Puccinia striiformis* f. sp. *tritici*.

SSRs Dataset	Regions	Frequency							Percentage (%) of all markers in its dataset						
		DNR*	TNR*	TTR*	PNR*	HNR*	COM^a^	Total	DNR*	TNR*	TTR*	PNR*	HNR*	COM^a^	Total
1113 new^b^	EXON	3	199	1	2	19	0	224	1.34	88.84	0.45	0.89	8.48	0.00	20.13
	INTRON	105	16	8	6	6	0	141	74.47	11.35	5.67	4.26	4.26	0.00	12.67
	PROMOTER	189	37	35	35	15	0	311	60.77	11.90	11.25	11.25	4.82	0.00	27.94
	INTERGEN	266	53	40	56	22	0	437	60.87	12.13	9.15	12.81	5.03	0.00	39.26
	Total	563	305	84	99	62	0	1113	50.58	27.40	7.55	8.89	5.57	0.00	100.00
105 poly^c^	EXON	0	12	0	0	0	0	12	0.00	100.00	0.00	0.00	0.00	0.00	11.43
	INTRON	17	1	1	1	1	0	21	80.95	4.76	4.76	4.76	4.76	0.00	20.00
	PROMOTER	22	6	2	0	1	0	31	70.97	19.35	6.45	0.00	3.23	0.00	29.52
	INTERGEN	31	4	1	3	2	0	41	75.61	9.76	2.44	7.32	4.88	0.00	39.05
	Total	70	23	4	4	4	0	105	66.67	21.90	3.81	3.81	3.81	0.00	100.00
30 reported^d^	EXON	1	17	0	0	0	2	20	5.00	85.00	0.00	0.00	0.00	10.00	66.67
	INTRON	0	0	0	1	0	0	1	0.00	0.00	0.00	100.00	0.00	0.00	3.33
	PROMOTER	3	1	1	0	0	1	6	50.00	16.67	16.67	0.00	0.00	16.67	20.00
	INTERGEN	1	2	0	0	0	0	3	33.33	66.67	0.00	0.00	0.00	0.00	10.00
	Total	5	20	1	1	0	3	30	16.67	66.67	3.33	3.33	0.00	10.00	100.00

* DNR, TNR, TTR, PNR, and HNR indicate di-, tri-, tetra-, penta-, and hexa-nucleotide SSRs, respectively.

^a^ COM indicate compound SSRs which containing more than one repeat type within 100bp.

^b^ The newly developed database containing 1113 SSR markers.

^c^ The 105 polymorphic SSR markers identified *in silico* using INDELs information.

^d^ The 30 SSR loci located by E-PCR with the previously reported SSR markers.

The SSRs containing tri-nucleotide repeats were the most frequent (>88.84%) among the five repeat types found in exon. Of the SSRs in exon, tri- and hexa-nucleotide SSRs that would not cause a frameshift accounted for 97.32%, while only 2.68% had potential to change the gene structure. We also calculated the frequency and percent of amino acids encoded by tri-nucleotide repeats in the corresponding 179 genes. These tri-nucleotide repeats encoded 11 kinds of amino acid (Table 5). Polar amino acids (QSTNG) accounted for 54.31% of those encoded, followed by acidic amino acids (ED,24.87%) and basic amino acid (HK,9.14%).

**Table 5:** Frequency and percent of amino acids encoded by tri-nucleotide motifs in their corresponding genes.

**Amino Acid**	**FREQ** *	**PCT** *	**Full Name**	**Property**
**Q**	38	19.29	Glutamine	Polar
**S**	33	16.75	Serine	Polar
**T**	18	9.14	Threonine	Polar
**N**	12	6.09	Asparagine	Polar
**G**	6	3.05	Glycine	Polar
**E**	27	13.71	Glutamic acid	Acid
**D**	22	11.17	Aspartic acid	Acid
**H**	14	7.11	Histidine	Basic
**K**	4	2.03	Lysine	Basic
**P**	17	8.63	Proline	Nonpolar
**A**	6	3.05	Alanine	Nonpolar

* FREQ: frequency; PCT: percentage (%).

**Fig 2:**
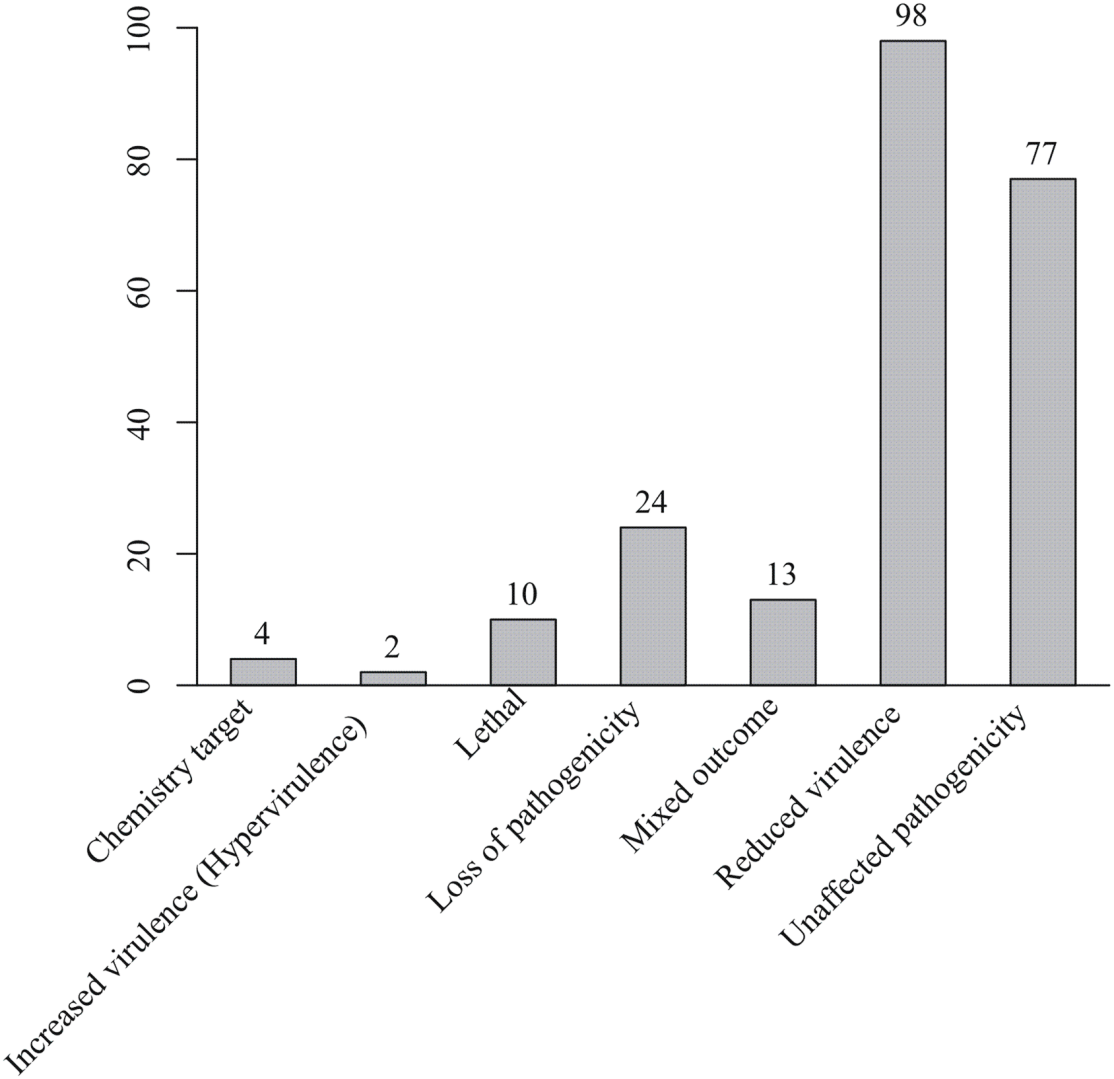
Functional categories of *Puccinia striiformis* f. sp. *tritici* genes closely linked with SSRs using the PHI database

A total of 972 SSR markers in the developed database mapped in the exon, intron, promoter regions and within 2kb downstream regions of 828 *Pst* genes. Among them, 104 SSR markers were closely linked with secreted proteins which might be involved in the plant and pathogen interactions. In addition, there were 268 markers distributed among 228 genes found to be homologs of genes with characterized functions in the PHI database. More than half of these genes were related to reduced virulence or loss of pathogenicity (Fig 2), indicating that the genes may play important roles in infection and development process of the stripe rust pathogen [45,46,49]. These SSR markers may therefore facilitate the genetic study of these genes in *Pst* populations. In contrast, only 10 out of 30 previously reported markers were linked to PHI homologous genes, and only 1 marker was linked to a secreted proteins. These results further indicated the usefulness of the database in studying *Pst*.

### 3.5 Identification of polymorphic SSR markers using INDEL variation

**Fig 3:**
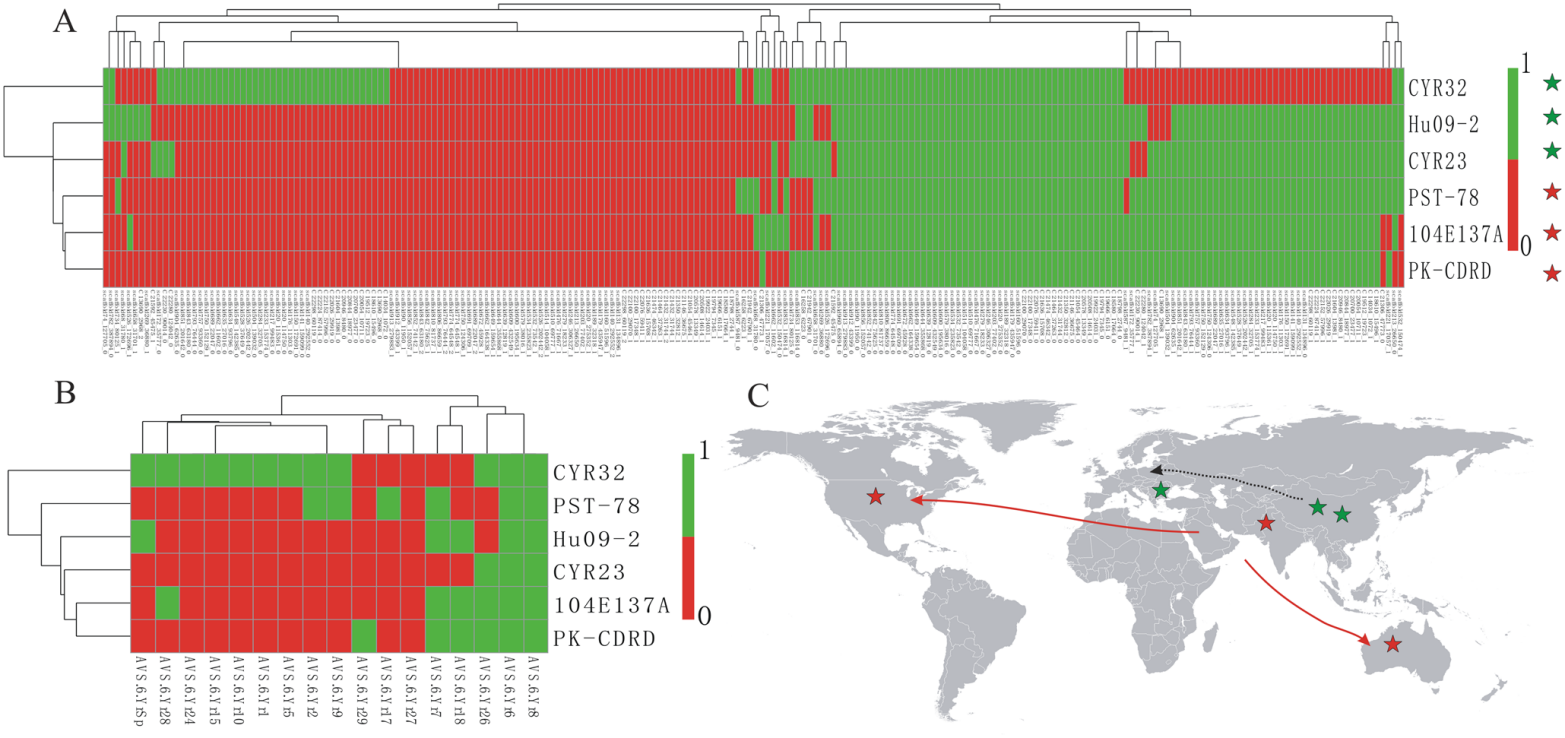
Relationships among six *Puccinia striiformis* f. sp. *tritici* (*Pst*) isolates. (A) Heat map and race relationship of six *Pst* isolates according to the INDEL variation in the 105 polymorphic SSRs using Pheatmap software. The presence and absence of bands were recorded as 1 and 0, respectively. (B) Heat map and relationship of the six *Pst* isolates according to their virulence on 17 differential cultivars downloaded from published paper using same method. The susceptible and resistant were recorded as 1 and 0, respectively. (C) Relative position of the six *Pst* isolates and their reported geographical origins.

In order to identify SSR markers with polymorphism, we identified INDELs present in the six isolates using GATK and Samtools software. As shown in S5 Fig, there are 13,143 concordance INDELs, 4,975 unique-to-GATK INDELs and 2,555 unique-to-Samtools INDELs. Because concordance INDELs were more robust than unique-to-method INDELs [42], we focused on concordance INDELs for the sake of accuracy. The identical sequence positions and nucleotide variations of the putative INDELs and of the SSRs directly revealed the presence of polymorphisms for 105 SSRs (S5 Table), accounting for 9.43% of the newly developed SSR markers. The tree based on these INDELs (Fig 3A) showed a similar topology to the virulence-based tree (Fig 3B) for the six isolates except PST-78 from the USA, which harbored INDEL polymorphisms similar to isolates from Pakistan and Australia but had a much different virulence pattern. Moreover, the INDEL-based tree showed some correlation with geographical origin. The tree suggested that PST-78 (USA), 104E137A (Australia), and PK-CDRD (Pakistan) had the same origin, and that CYR23 (China), CYR32 (China), and Hu09-2 (Hungary) might have another origin (Fig 3C), which is consistent with a recent worldwide population structure study of *Pst* [32].

The SSRs with di-, tri-, tetra-, penta-, and hexa-nucleotide motifs accounted for 66.67%, 21.90%, 3.81%, 3.81%, and 3.81% of these 105 SSRs, respectively (Table 3). Of the 105 polymorphic SSR markers, 12 markers were in exons, 21 in introns, 31 in promoter regions, and 41 in intergenic regions (Table 4). Among them, 11 were associated with secreted proteins, while 24 were associated with pathogenicity-related genes. Moreover, only marker scaffold532-150474 shared a locus with a previously reported marker (SUNIPst09-40) (S2 Fig). Therefore, there are 104 markers would amplify loci which are not targeted by the previously reported 84 SSR markers.

### 3.6 SSR markers validated for quality and polymorphism

**Fig 4:**
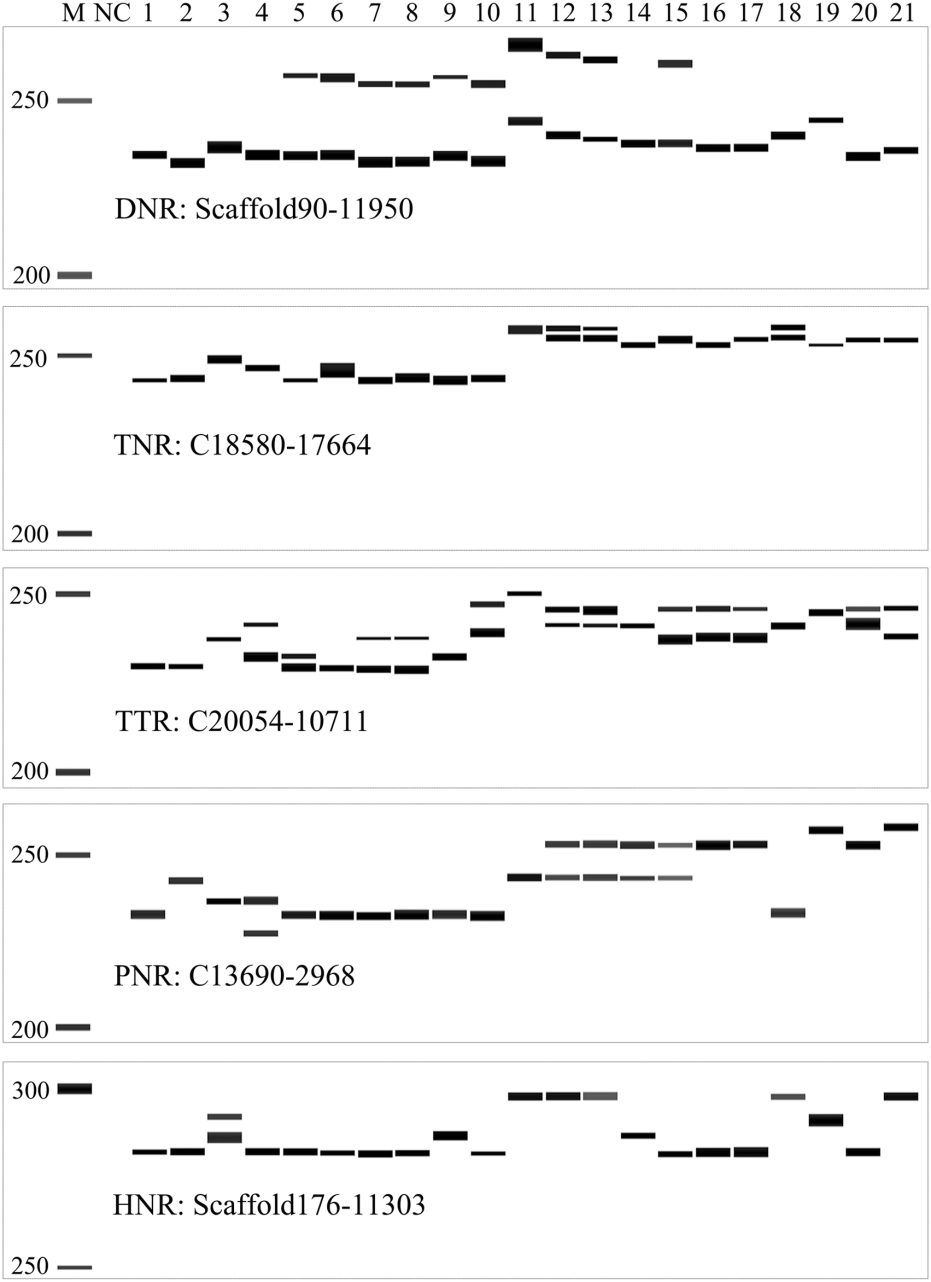
Experimental validation of five representative SSR markers in 21 *Puccinia striiformis* f. sp. *tritici* isolates. Gel images are shown from the analyses performed with the QiaxcelScreenGel software. Lane M, a size marker; Lane NC, a negative control; and Lanes 1–21, the PCR products of 21 corresponding isolates. DNR, TNR, TTR, PNR, and HNR indicate di-, tri-, tetra-, penta-, and hexa-nucleotide SSRs.

**Fig 5:**
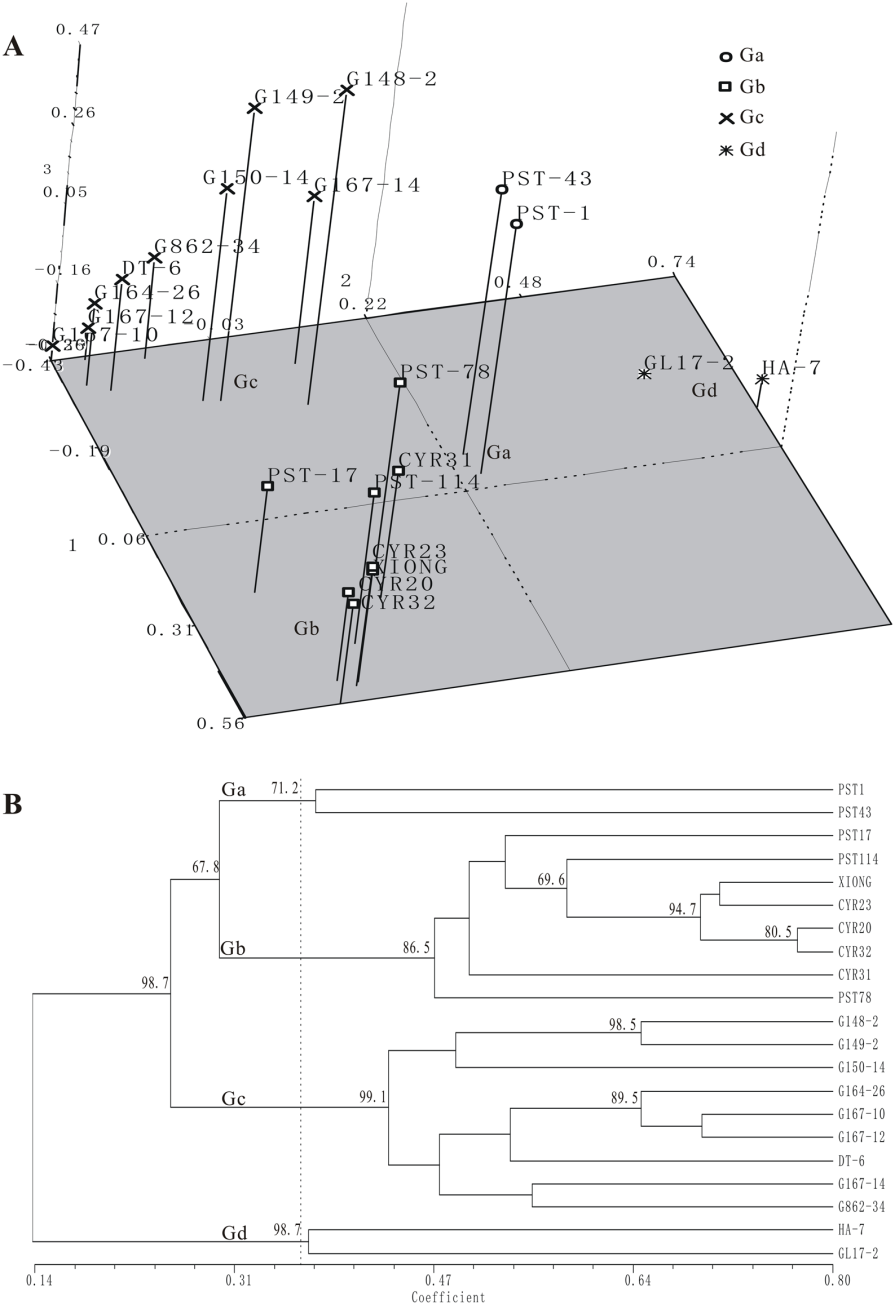
Relationship of 21 isolates based on genotypes of 82 experimentally validated SSR markers. (A) Principal component analyses. (B) Dendrogram showing the similarities based on the UPGMA; the number at each branch shows the bootstrap value with 1,000 replications.

After excluding the SSR marker scaffold532-150474 sharing the locus which could be amplified with a previously reported marker SUNIPst09-40, the other 104 *in silico* polymorphic SSR markers were experimentally validated with 21 isolates (Table 1). Eighty-four of the markers generated specific bands, while 20 primer pairs failed to produce stable or clear bands due to a lack of sequence specificity in the genomic DNA samples. Of the 84 primer pairs, 82 revealed allelic difference among the 21 isolates tested (Fig 4, details are presented in S6 Table). For these polymorphic SSR markers, the motif length ranged from 14 to 42bp with an average of 19.48bp. Repeat numbers ranged from 4 to 15 units with an average of 7.96. In total, 477 alleles were amplified, with a range of 2–12 alleles at an average of 5.82 alleles. Product sizes ranged from 116 to 307bp. Polymorphic information content (PIC) values ranged from 0.17 to 0.88 with an average of 0.71. Observed heterozygosity (Ho) ranged from 0.00 to 0.95 with an average of 0.31, and expected heterozygosity (He) ranged from 0.17–0.88 with an average of 0.71. Six markers were dominant in these 21 isolates, and the other 76 markers were co-dominant. The relatively high PIC and relatively low observed heterozygosity could be due to a higher conservation of these SSR loci among the 21 isolates, achieved by selecting unique flanking sequences in multiple *Pst* genomes. Based on the molecular genotypes of the 82 polymorphic SSR markers, both principal component analysis and cluster analysis separated these 21 isolates into four groups (Fig 5): group Ga with only two isolates from the US; group Gb with isolates from the US, China and Hungary; group Gc with isolates from Xinjiang, Qinghai and Tibet in China; and group Gd with isolates from Xinjiang and Gansu in China. This result was consistent with previous report that the *Pst* populations of China and the US in general evolved independently [31]. These results indicated that newly developed SSR markers were informative and useful; approximately 80.77% of the primers in our SSR database were effective. It was highly efficient (97.62%) to identify polymorphic SSR markers using the INDEL data based on whole genome re-sequencing of multiple isolates.

## Discussion

SSRs are abundant and dispersed throughout *Pst* genomes. A total of 4,792 SSR loci were identified from six *Pst* isolates from five countries. This number is much larger than the 1,889 loci found in another genome-derived SSR identification [24] using the PST-130 sequence that cover only about 60% of the *Pst* genome [33]. The much larger number was achieved through the much better coverage of the sequence data from six isolates. This abundance of SSRs in *Pst* is neither the highest nor the lowest reported for fungi [50]. In the *Pst* genome, the di- and tri-nucleotide SSR motifs were more abundant than tetra-, penta-, and hexa-nucleotide motifs. SSR densities for different types of motifs varied from 51.04 to 411.99 kb. Moreover, we found that the AG/CT and ATC/ATG repeats were the most abundant di- and tri-nucleotide SSRs, respectively, in *Pst*. The abundance of AG/CT motifs is similar to the reports for other fungi, but our finding of abundant ATC/ATG motifs in *Pst* is different from other fungi [17,50], suggesting that *Pst* shares some common features in SSR loci formation and structure with other fungi, but also has its unique features. SSRs containing tri-nucleotide repeats were the most frequent (>88.84%) among the five repeat types found in exon, which was consistent with other reports [51–54]. Such phenomenon maybe due to the selection against frameshift mutations in coding regions [55]. More studies on the exon tri-nucleotide repeat SSR loci in comparison with other SSR loci may lead to a better understanding of the *Pst* evolution.

Because of abundance, easy to use, and highly polymorphic, SSR markers have been widely used in population studies of *Pst* [25,28,29,32]. However, the number of SSR markers publicly available prior to this study was limited [20–24]. In this study, we developed a database containing 1,113 SSR markers, which have unique flanking sequences across 6 *Pst* genomes. Moreover, the 1,113 markers were mostly abundant in intergenic regions. Compared with most of previously reported EST-SSRs developed based on EST libraries [21–23], these SSRs cover more non-transcribed regions, which can be advantageous in studies focusing on evolutionary or migratory studies of the pathogen without much selection. The 104 SSR markers identified among the six isolates were found to be closely linked with secreted proteins, should be useful makers to tag potential avirulence loci. In addition, 268 markers were found to be linked with 228 proteins that were homologous to proteins in the PHI database [45,46,49] and 53.51% of them were annotated as proteins related to reducing virulence or losing pathogenicity [49]. These results indicate that these markers may provide information of genetic variations of these genes and can be used for studying genes involved in infection and development process of *Pst*. For example, these markers could be used to study the associations between these genes and pathogenic traits in *Pst* population [56–59]. Of the 104 SSR markers used in experimental validation, 82 primer pairs were polymorphic among 21 isolates tested, and we determined the genetic relationships of the isolates using markers. Therefore, SSR markers identified in this study should be useful in a variety of applications, such as studying of population structures, mapping avirulence genes or genes for other important traits, and tagging or monitoring particular populations, virulence, and pathotypes of the pathogen.

*Pst* populations possess a high genetic diversity and virulence variability [7], giving them the ability to counteract the specific resistance genes in wheat cultivars [5,8,9]. Therefore, monitoring the pathogen populations is important for control of the disese. Traditionally, the *Pst* populations are monitored through virulence surveys [60] or just using a small number of molecular markers [28–31]. The availability of more markers can differentiate isolates better and improve our understanding of the genetic diversity and population structure of *Pst* in various agro-ecosystems. The population dynamics of *Pst* may provide useful information for rational deployment of resistance gene in wheat cultivars.

It is highly efficient to identify potential polymorphic markers by finding identical sequence positions and nucleotide variations of SSRs and of INDELs using whole genome re-sequencing of multiple isolates. In the present study, we identified 13,143 concordance INDELs using GATK and Samtools software across six *Pst* isolates from different countries. Then, 105 polymorphic SSR markers were identified *in silico* using proof of short tandem repeat INDELs from 1,113 SSR loci. From 84 markers generated specific bands, 82 primer pairs (97.62%) were polymorphic. In comparison, experimental testing those directly from potential markers usually results in less than 20% polymorphic markers [20–24]. Therefore, the strategy used in the present study further makes the development of useful SSR markers through the approach of genome re-sequencing of multiple isolates more efficient.

In conclusion, whole genome re-sequencing of multiple isolates is efficient for the developing SSR markers. Using the approach, we identified 4,792 SSR loci at an average interval of 22.95 kb for the stripe rust pathogen. We developed a database containing 1,113 SSR markers and validated the polymorphisms of 82 markers using 21 isolates. The SSR markers should be useful in various studies for a better understanding of the pathogen in order to more effectively control strip rust. The approach and methods shown in this study are applicable in developing SSR markers for any other organisms.

## Supporting Information

S1 Fig Flow chart describing the main steps used to develop SSR markers in *Puccinia striiformis* f. sp. *tritici*. (TIF)

S2 Fig Genomic location of 34 previously reported SSR markers with single E-PCR primer binding site. (A) Location of newly developed SSR markers with *in silico* polymorphic (in green color). (B) Location of 34 previously reported SSR markers (in blue color). (C) Location of newly developed SSR markers in the database containing 1,113 SSR markers (in red color; location of 14 shared loci between previously reported and newly developed SSR makers were highlighted in blue color).
(TIF)

S3 Fig Genomic location of 23 previously reported SSR markers with two E-PCR primer binding sites. (A) Location of newly developed SSR markers with *in silico* polymorphic (in green color). (B) Location of 23 previously reported SSR markers (in blue color). (C) Location of newly developed SSR markers in the database containing 1,113 SSR markers (in red color). Links were used to connect the two primer binding sites of a marker.
(TIF)

S4 Fig Genomic location of five previously reported SSR markers with three E-PCR primer binding sites. (A) Location of five previously reported SSR markers (in blue color); (B) Location of newly developed SSR markers in the database containing 1,113 SSR markers. Links in same color were used connect the three primer binding sites of a marker.
(TIF)

S5 Fig SSRs indentified with INDELs called by GATK and Samtools. GATK-INDELs represents the INDELs called by GATK; Samtools-INDELs represents INDELs called by Samtools; and SSR_Database represents the 1,113 newly developed SSR markers.
(TIF)

S1 Table The number of SSRs in different repeat types in the *Puccinia striiformis* f. sp. *tritici* genome. (XLSX)

S2 Table The 1,113 SSR markers with *in silico* specificity. Lines (font in red) indicate loci shared with the reported SSR markers.
(XLSX)

S3 Table The 84 reported SSR markers downloaded from 4 published papers. (XLSX)

S4 Table E-PCR primer binding sites and genomic locations of the 84 reported SSR markers. (XLSX)

S5 Table The 105 polymorphic SSR markers and their corresponding INDELs. (XLSX)

S6 Table Details of the 82 SSR markers experimentally validated in 21 *Puccinia striiformis* f. sp. *tritici* isolates. Including primer sequences, annealing temperature, number of alleles, product size range (bp), polymorphic information content (PIC), observed heterozygosity (Ho) and expected heterozygosity (He).
(XLSX)
