# Supplementary figures and images for: Genome-Wide Analysis of Simple Sequence Repeats and Efficient Development of Polymorphic SSR Markers Based on Whole Genome Re-Sequencing of Multiple Isolates of the Wheat Stripe Rust Fungus
Source: PLoS One. 2015 Jun 12;10(6):e0130362. doi: 10.1371/journal.pone.0130362 (PMC4467034; doi:10.1371/journal.pone.0130362)

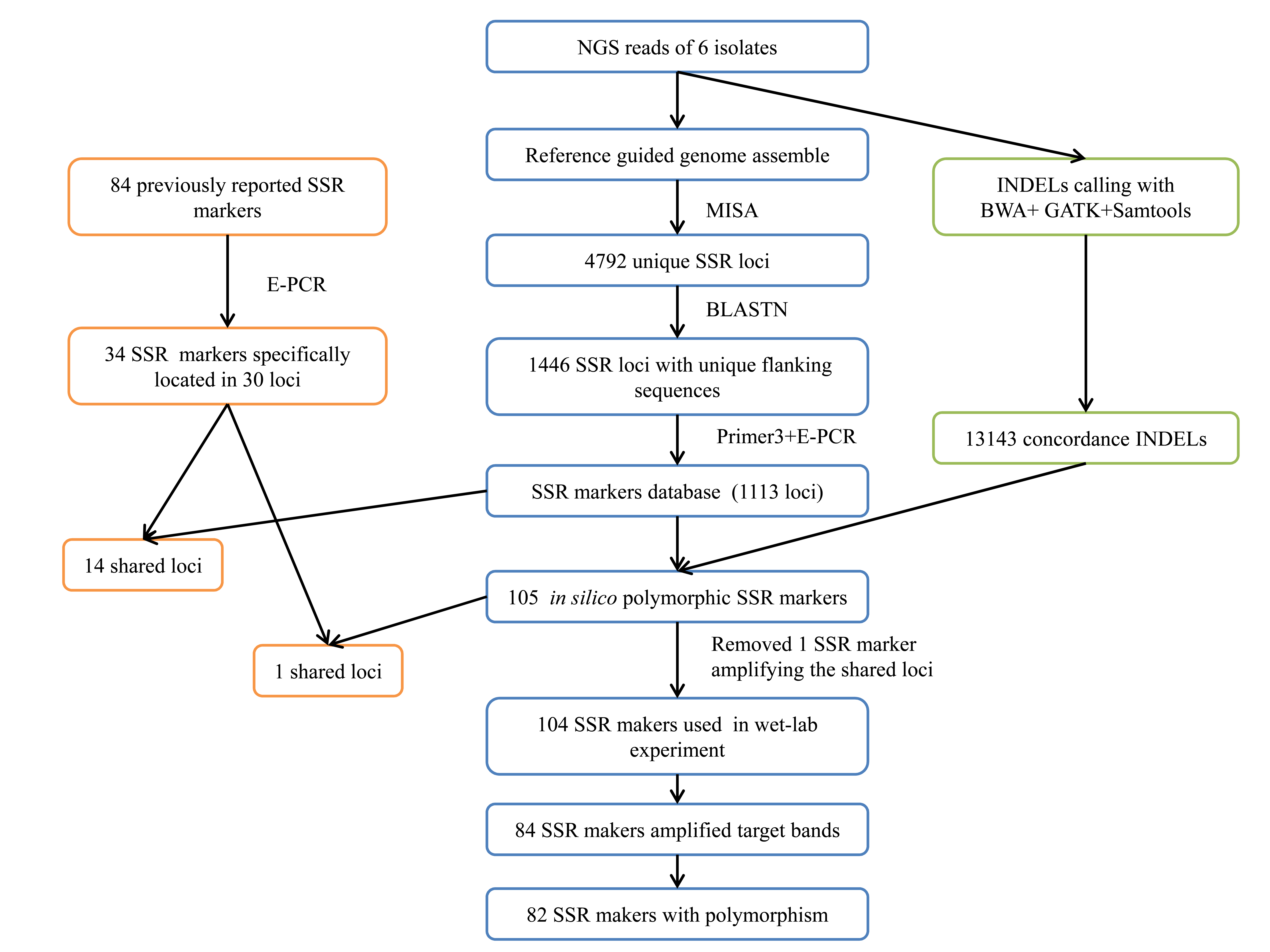

Supplement: S1 Fig — (TIF) [file pone.0130362.s001.tif]

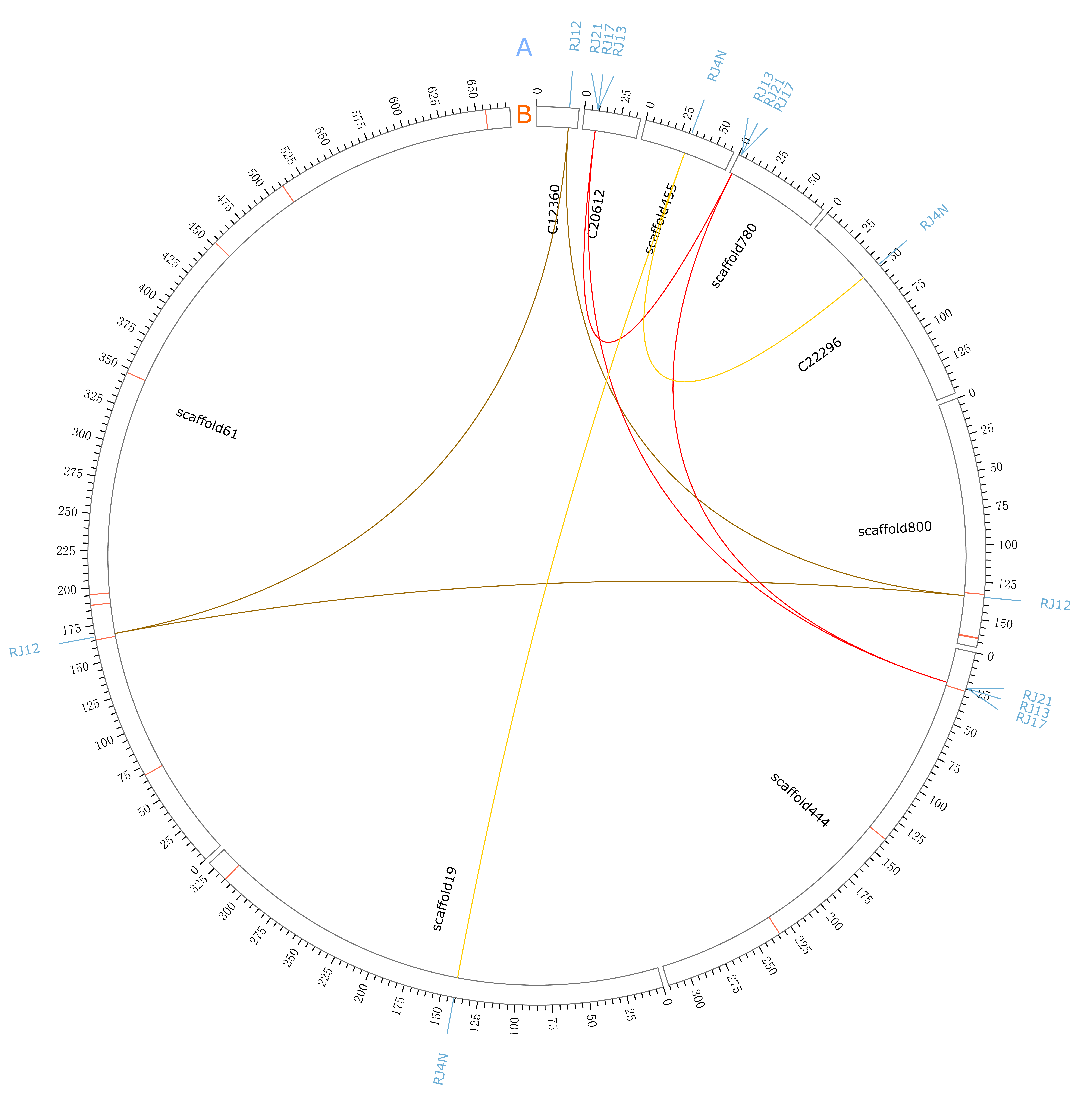

Supplement: S4 Fig — (A) Location of five previously reported SSR markers (in blue color); (B) Location of newly developed SSR markers in the database containing 1,113 SSR markers. Links in same color were used connect the three primer binding sites of a marker. (TIF) [file pone.0130362.s004.tif]

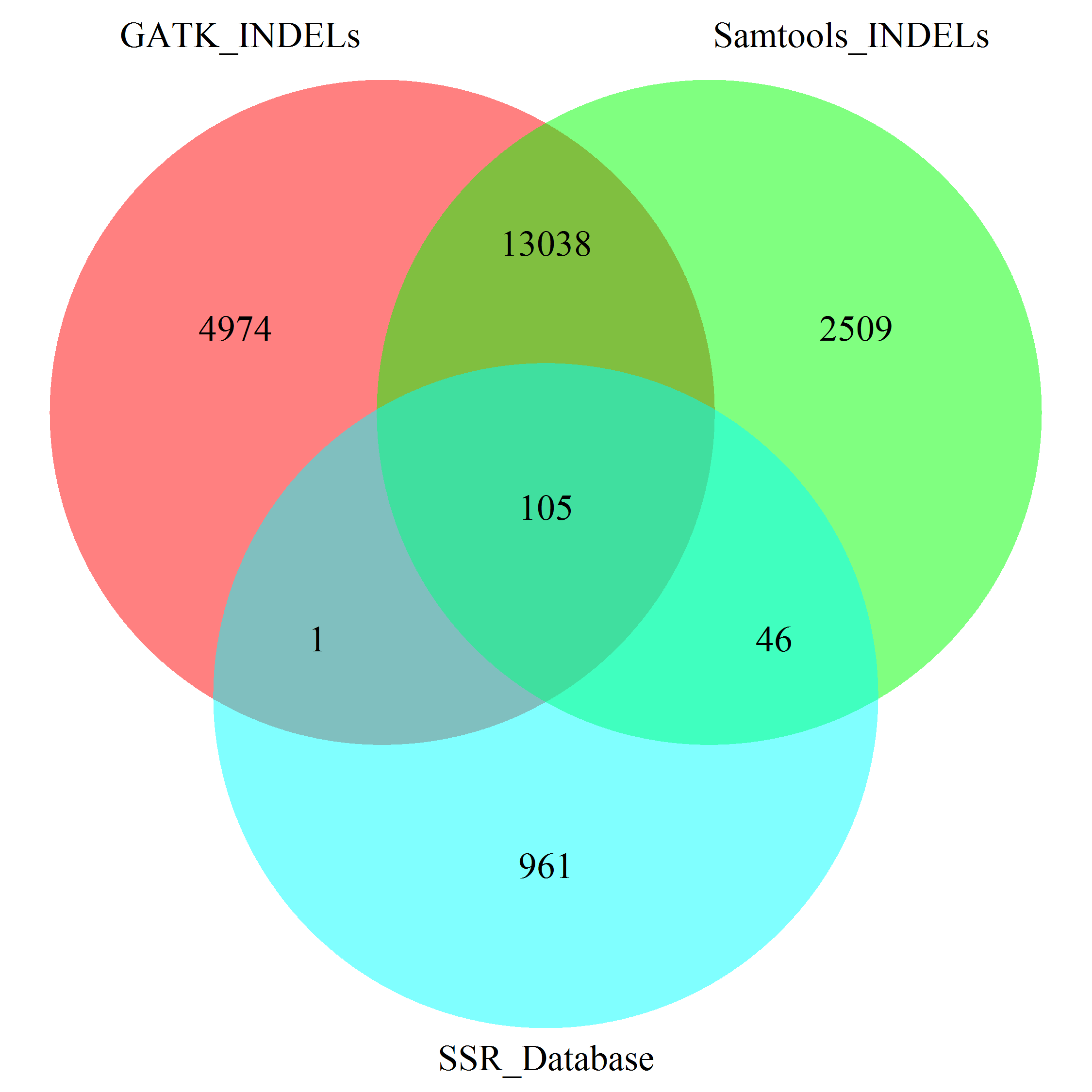

Supplement: S5 Fig — GATK-INDELs represents the INDELs called by GATK; Samtools-INDELs represents INDELs called by Samtools; and SSR_Database represents the 1,113 newly developed SSR markers. (TIF) [file pone.0130362.s005.tif]
